# Bactericidal efficiency of micro- and nanostructured surfaces: a critical perspective

**DOI:** 10.1039/d0ra08878a

**Published:** 2021-01-13

**Authors:** S. W. M. A. I. Senevirathne, J. Hasan, A. Mathew, M. Woodruff, P. K. D. V. Yarlagadda

**Affiliations:** Science and Engineering Faculty, Queensland University of Technology (QUT) Brisbane Qld 4000 Australia jafar.hasan@qut.edu.au jafarhasan13@hotmail.com; Institute of Health and Biomedical Innovations 60 Musk Ave. Kelvin Grove Qld 4059 Australia

## Abstract

Micro/nanostructured surfaces (MNSS) have shown the ability to inactivate bacterial cells by physical means. An enormous amount of research has been conducted in this area over the past decade. Here, we review the various surface factors that affect the bactericidal efficiency. For example, surface hydrophobicity of the substrate has been accepted to be influential on the bactericidal effect of the surface, but a review of the literature suggests that the influence of hydrophobicity differs with the bacterial species. Also, various bacterial viability quantification methods on MNSS are critically reviewed for their suitability for the purpose, and limitations of currently used protocols are discussed. Presently used static bacterial viability assays do not represent the conditions of which those surfaces could be applied. Such application conditions do have overlaying fluid flow, and bacterial behaviours are drastically different under flow conditions compared to under static conditions. Hence, it is proposed that the bactericidal effect should be assessed under relevant fluid flow conditions with factors such as shear stress and flowrate given due significance. This review will provide a range of opportunities for future research in design and engineering of micro/nanostructured surfaces with varying experimental conditions.

## Abstract 2

Micro/nanostructured surfaces (MNSS) have shown the ability to inactivate bacterial cells by physical means.

## Graphical abstract

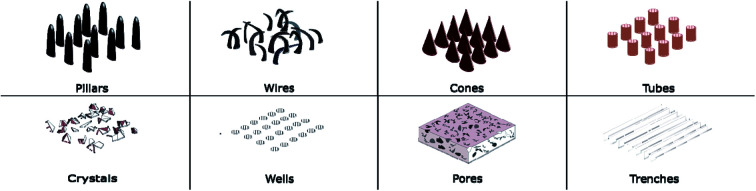

## Introduction

Bacterial cells can adhere onto surfaces, colonise, and lead to biofilm formation. Adhesion of bacteria can occur on various types of surface such as on human tissues, or metallic or polymeric surfaces. Adhered bacteria secrete extracellular polymeric substances (EPS) into their environment, and form biofilms.^1^ EPS provide the structural support to the biofilms which are highly resistant to anti-septics, antibiotics, and immune killing.^2,3^ Biofilms can be formed at solid to liquid, solid to air, and liquid to air interfaces.^2^ This shield of biofilm is physically strong and has high viscoelasticity, making the removal of the biofilm from the adhered surface extremely difficult.^1,4^ These biofilms affect various sectors ranging from healthcare to engineering sectors. For example biofilms can cause infections,^5^ block filtration mechanisms,^6^ block aviation fuel systems,^7^ reduce efficiency of heat exchanger channels,^8^ increase drag resistance which consequently increases fuel consumption of marine vessels,^9^ and also increase the heat load of buildings.^10^ Bacteria acquiring antibiotic drug resistance is a big issue faced by the health sector in treating bacterial infections.^11^ Bacterial infections in bioimplants cause numerous problems such as bioimplant failure that often leads to the requirement for revision surgery, and the associated requirement of prolonged hospitalisation, or even mortality.^11–13^ Though many surface-based strategies are bactericidal, yet there are some antibiofouling strategies which include bacterial repulsive, slippery, and highly hydrophobic surfaces. Numerous remedies are taken to mitigate the issue of bacterial colonisation and associated subsequent consequences. Use of bactericidal and bacteria repelling chemical agents, such as antibiotics, and antiseptics is a widely used method. Coating surfaces with bio repellents is another approach that has become popular.^14,15^ In recent years, nanoparticle coatings have been developed to prevent bacterial attachment onto solid surfaces.^16^ Functionalised surfaces that can release bactericidal agents is also another method that has been tested extensively.^17^ When attached to a surface, some bacteria are known to be resilient to antibiotics which would otherwise be effective if they were suspended in a liquid medium.^18^ This provides challenges for the conventional methods in mitigating bacterial adhesion and related issues. Therefore, alternatives for preventing bacterial colonisations and infections are in high demand.

It has been discovered recently that on certain natural surfaces, bacteria are dying more than usual. Nanostructures on those natural surfaces such as cicada wings,^19–21^ dragonfly wings,^22^ and gecko skin^23,24^ possess an ability to lyse bacterial cells. Mostly these surfaces are superhydrophobic and have the ability of self-cleaning. It has been demonstrated that the nanopillars or nanostructures on these surfaces kill the bacteria by membrane rupture rather through a chemical mechanism^20,25^ as these surfaces do not have an inherent chemical coating that is bactericidal.^19^ On most of the studied insect wings surfaces, nanopillars have a height of 200–300 nm, 40–100 nm diameter at the tip, and 100–200 nm spacing between the pillars.^19,22,26^ While many of these nanoscale features have patterned structures such as those on cicada wings,^27^ random nanofeatures are also present such as those on dragonfly wings^22^ and gecko skin,^28^ which have sometime better bactericidal efficiencies.^19^ Discovery of bactericidal nanostructured bio-surfaces have created promising alternatives to mitigate bacteria related issues such as infections and biofouling.

Bioimplants are essential in orthopaedics, dentistry, tissue engineering, and reconstructive surgery.^29^ Cytocompatibility of the material is another important property that is vital for success of these implants. A non-bioactive implant would enhance the formation of fibrous tissue leading to implant failure. Eukaryotic cell growth on implant surfaces depends on the chemistry, wettability, surface roughness and topography of the surface.^30^ In many cases, bactericidal nanostructured surface does not facilitate eukaryotic cell growth and proliferation. Therefore, it is important to test the efficacy of micro/nanostructured topography on a bioimplant surface against both bacteria and mammalian cell adhesion and proliferation. Understanding of bactericidal properties of natural surfaces and eukaryotic cell adhesion behaviour on substrates has inspired researchers to develop and fabricate novel artificial bactericidal surfaces that bio-mimics natural bactericidal surfaces on implant surfaces.^31–34^ Similar to its application in biomedicine, micro/nanostructured surfaces (MNSS) also hold its potential in other areas such as anti-biofouling ship hulls, industrial pipelines, food processing, furniture and public spaces. However, investigation of MNSS in these areas is still in its infancy.

Generally, bacteria are categorised into several types based on their anatomy or motility, and such bacterial types have responded to MNSS differentially. Gram stain testing differentiates bacteria based on their response to staining. Gram-positive bacteria have the cell wall with thick layers of peptidoglycans with an outer lipid membrane, and on the other hand, Gram-negative bacteria contains a thin peptidoglycan layer with no outer lipid membrane.^35^ Hence, Gram-positive bacteria are considered to be more resilient to the MNSS compared to the Gram-negative counterparts.^20,21^ However, high resilience of Gram-positive species of bacteria to surface-modified substrates is disputed with observations from recent studies,^36–40^ discussed later in this review. Another classification of bacteria is based on their motility. Motile bacteria are capable of active motion by self-propulsion, while movement of non-motile bacteria are due to the passive motion influenced by the surrounding media.^41^ The motile species of bacteria use their extracellular appendages to obtain motion.^42^ Interestingly the bactericidal efficacy (BE) of MNSS has been reported to be more pronounced on motile bacteria.^26,43^ However, no studies on MNSS have defined or investigated the influence of bacterial types by including both bacterial motility as well as Gram stain classification. Therefore, a comprehensive study on the effect of bacterial type on the bactericidal property of MNSS would be highly valuable in designing antibacterial surfaces. It is to note that bacterial classification is also based on the different shapes of bacteria such as coccus, bacillus, coccobacilli, or spirilla. While their individual influence on the BE of MNSS seem significant but in this study, bacterial shape has been largely grouped under Gram positive or Gram-negative counterparts or grouped under motility. This has been done to narrow down the recent limited literature for a better understanding and future design of BE on MNSS.

In view of the above aspects, this review has been undertaken to assess the state-of-the-art bactericidal MNSS. Various important aspects of bactericidal MNSS which have never been analysed are reviewed here.

In this review, the prominent issues that require current attention for future research and development in MNSS design and translating bactericidal MNSS to clinical or industrial applications, are presented. These include (i) factors that affect bactericidal activity, (ii) bacterial viability measurement methods on the MNSS and (iii) an in-depth introspection of bacteria under flow conditions on MNSS and in microfluidic devices. This review also highlights important aspects of evaluating bactericidal performances of MNSS *via* critically analysing currently used BE quantification methods.

## Classification of bactericidal MNSS based on architecture

**Fig. 1:**
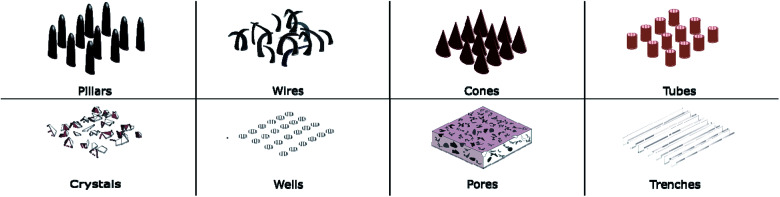
Different nano structural features are fabricated on substrates. Pillars, wires, cones, tubes, and crystals are categorised as “protrusion features”, while wells, pores and trenches are categorised as “recess features”.

Diverse types of micro/nanoscale features have been engineered on a wide range of materials. These features can be divided into two major categories, based on their geometry as either protrusion or recess types. Protrusion type features are pillars, wires, spikes, tubes, cones, and crystals while pores, trenches and wells can be categorised as recess type features. These are illustrated in Fig. 1. Protrusion type features can be further identified as two different subcategories, with low and high aspect ratio features. Generally, pillars, wires, spikes, and cones have high aspect ratios while tubes and crystal type features have low aspect ratios.

The stronger the influence of the nanoscale features on the bacteria, the higher is the chance of bacterial lysis or death. Bactericidal activity influenced by surface topography is heavily dependent on the nanoscale architecture, among them pillars, wires, cones and crystals have generally shown a stronger affect than other surface architectures.

## Factors affecting bactericidal property of MNSS

Factors such as feature shape, size, substrate surface hydrophobicity, roughness and bacterial species influence bacterial adhesion and viability on solid substrates. However, micro/nano-topography of the surfaces of MNSS have shown to be most critical in determining the BE.^26,31,33,36,39,44–47^ In addition, BE has been reported to be influenced by a number of other parameters such as the type of medium used for bacterial cell incubation,^48^ bacterial inoculum concentration,^49,50^ incubation time,^39,45,51,52^ strain of bacteria.^26,45,53^ Despite the popular belief that micro/nanoscale topographies are more effective against Gram-negative strains, numerous studies have also reported higher or equivalent BE of MNSS against Gram-positive strains.^26,36–39,51,54^ For example, in a study, Jaggessar *et al.*, fabricated six different TiO_2_ nanopillars on titanium substrates and studied their bactericidal effect against both Gram-negative (*Pseudomonas aeruginosa*) and Gram-positive (*Staphylococcus aureus*) bacteria.^36^ All six surfaces tested for bacterial viability, which showed a higher bactericidal rate on Gram-positive than on Gram-negative strains. However, the nanostructure investigated in this research is nano-wire type with some interconnection between features rather than non-connected or self-standing pillar structures which most of other studies had used. Moreover, these nanowires were sharper with diameters ranging from 17–42 nm, compared to most of pillar type nanostructures reported in literature that has pillar tip diameters above 100 nm.^28,38,44,45,54–57^ Several other reports also confirmed that thinner features demonstrate high bactericidal effect on Gram-positive species compared to Gram-negative strains. For an example, 20–40 nm diameter titanium nano tubes exhibited 51–60% BE against *S. aureus*,^58^ and silicon pillars with 21 nm diameter on *S. aureus* was 90–97% (ref. 37) and 50 nm silicon pillar on *Bacillus subtilis* was 65% (ref. 37) and 90%.^39^ Whilst, many of the large diameter features failed against Gram-positive species, pillar diameters as large as 490 nm have been successful on Gram-negative species, *R. capsulatus* and *E. coli*.^38^ However, it is noteworthy that there had been differential BE against bacterial species of the same Gram-stain type. For an example, titanium nano-wire structure with 100 nm feature diameter exhibited BE > 80% against Gram-negative *Escherichia coli*, it only showed BE ≈ 5% against Gram-negative *Klebsiella pneumoniae*.^26^

Correlating hydrophilicity of MNSS to its bactericidal effect has also been a challenge. Surface treatment methods can be used to alter the surface wettability to fabricate surfaces with different hydrophobicity but with the same nanotopography.^36,37^ Although hydrophobic surfaces have exhibited BE > 75%, no direct relationship has been established between hydrophobicity and strain dependent bacterial death. Hydrophobic MNSS displayed BE > 80% against both Gram-positive,^37,39,40,57^ and Gram-negative strains^26,28,37,39,44,53^ of bacteria. Similarly no correlation is evident on hydrophilic MNSS as some hydrophilic surfaces were effective against Gram-positive,^38,51^ while some were effective against Gram-negative strains,^37,38,45,51,53^ and some effective against both types.^37,38,51^ Despite high bactericidal effect by some hydrophobic surfaces and hydrophilic surfaces as above, certain hydrophobic^28,55^ as well as hydrophilic^36,46,55,58–61^ MNSS are reported with low or no bactericidal effect. Most of unsuccessful substrates were titanium,^36,46,58–60^ and features were pores,^59^ tubes,^58^ or crystals.^60^ Titanium nano-pillar structures fabricated by (ref. 40 and 46) were 50 nm and 33 nm in pillar tip diameter respectively. While the former deemed highly efficient (BE > 80%) against *S. aureus*, the latter failed against the same species of bacteria. Despite the similar nanostructure, the hydrophobicity of the two surfaces were largely different, with former being hydrophilic and the latter being hydrophobic. Similarly, the same silicon nano-pillar structure fabricated two substrates with varying hydrophobicity has demonstrated differential BE against the same bacterial species.^55^ BE of both the surfaces were less than 50%, but the hydrophilic surface was less efficient than the hydrophobic counterpart.

Hydrophobicity of the surface may have a degree of influence on the bactericidal effect, but there are likely other factors that govern the overall responses, including physio-chemical and mechanical properties that may influence the bactericidal activity. Hence, an in-depth analysis is highly essential to identify effects of feature shape and size, substrate surface characteristics, bacterial characteristics on success of bactericidal MNSS.

## Measuring bacterial viability on MNSS

The most common protocol to quantify BE of MNSS is incubate bacteria on the substrate and enumerate the live and dead cells. Incubation of cells are done under static conditions. The next subsections discuss this BE quantification protocol (static incubation) in detail.

### Static bacterial viability quantification protocol

**Fig. 2:**
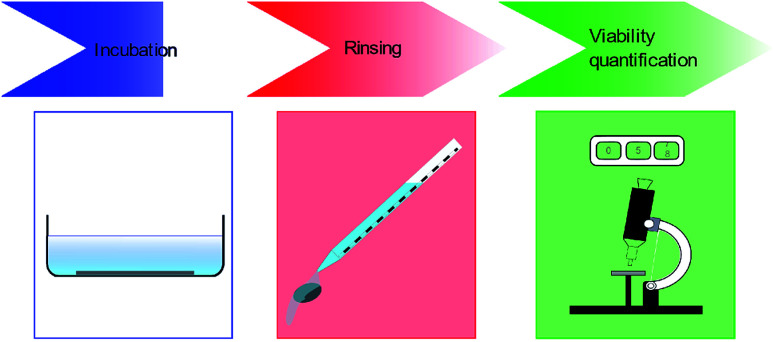
Classically, bacterial cell viability quantification protocols have three main steps. First the bacteria are incubated on MNSS substrate with substrate immersed in bacterial suspension, followed by a rinsing step to remove any non-adhered bacteria. Finally, BE is calculated either by live dead analysis or CFU measurements.

Bacterial viability quantification on substrates involve three major steps. Firstly, the bacteria are incubated on the MNSS. Secondly, the substrates are rinsed to remove any non-adhered or weakly adhered cells from the substrate. Finally, the number of live and dead cells attached to the surface are quantified using various techniques. Details of these 3 steps are depicted in Fig. 2, and discussed in detail in following sub sections.

### Incubation of bacteria on substrates

Static protocols are widely used to assess the viability of bacteria on MNSS. The major similarity of protocols used for these studies is that bacteria are incubated on substrates in a contained space with constant amount of growth media in static or agitated conditions.^26,28,31,33,36–40,44–48,51,53–56,58–60,62–64^ Phosphate buffered saline solution is a commonly used medium to make bacterial suspension. The substrate is immersed in bacterial suspension within a container such as in a microwell plate. A finite amount of growth medium is supplied to this container and generally replenishing of growth medium is not done. The substrate immersed in bacterial suspension^26,31,44–48,65^ or aliquot of bacterial suspension placed on substrate,^33^ are either kept stationary, or mechanically agitated. Incubation duration varies largely between studies from 1 to 18 hours. The incubation of bacteria in static medium will be referred in this article as static-condition.

### Rinsing substrate for removing non-adhered bacteria

For the viability experiment, a common step is to rinse the substrates to remove non-adhered or weakly-adhered bacteria from the surface before conducting quantification test such as live dead analysis or colony counting.^31,33,37,38,40,45,47,51,53,55,56,59,63,66–85^ Commonly used rinsing methods are summarised in Table 1. Strikingly, the volumes or flowrates used in the rinsing step of these studies are unspecified, and the effect of stresses exerted on the loosely attached bacterial cells have not been accounted for. Later in this review, the influence of fluid shear stress on detachment of bacterial cells has been discussed. If the threshold of bacterial adhesion strength is exceeded by the fluid flow, bacterial cells get advected even if the cells are strongly attached. Without undermining the significance of the previously published reports, this rinsing step adds an uncertainty to the process of bacterial viability enumeration on substrates.

**Table 1:** Rinsing methods used before bacterial viability quantification

Description of method	Ref.
Substrates washed by flowing sterile distilled water of unspecified flowrate	33, 37, 51 and 66–68
Substrates rinsed by flowing sterile PBS of unspecified flowrate	40, 45, 56, 59, 69–77 and 85
Substrates bath-sonicated in sterile PBS	53
Bacterial suspension was removed by aspiration, and substrates washed with PBS	78
Substrates rinsed by flowing Tris–HCl buffer of unspecified flowrate	55, 63, 79 and 80
Substrates immersed in Tris–HCl buffer, and passed back and forth several times	31 and 47
Substrates rinsed by flowing NaCl solution of unspecified flowrate	81–83
Substrates rinsed by flowing TBS of unspecified flowrate	38
Substrates rinsed by flowing fresh LB (Miller) broth of unspecified flowrate	84
Substrates vortex-stirred in PBS	60

### Quantification of bactericidal efficacy of MNSS

BE is mostly used as a measurement of antibacterial performance of MNSS. Typically, two main protocols have been adopted by researchers to quantify the BE of cells on MNSS. (i) Colony forming unit (CFU) measurements and (ii) live dead analysis. The definition of BE slightly varies between these methods. In the first method, bacterial cells adhered to the substrate are removed from the substrate and are incubated, and then the number of colonies are counted.^39,57^ In most of the studies, a control sample is used to enumerate the live bacterial colonies. The bactericidal efficacy is calculated by taking the percentage reduction in CFU of sample and a control,^39^ which is closely derived from International Standards Organisation (ISO) standard ISO 22916:2011.^86^ This method is based on several fundamental assumptions. Firstly, it is assumed that all bacterial cells that were adhered onto the substrate is removed from it and taken into the suspension. Secondly, if the antibacterial surfaces do not contain any leaching agents and only possess contact-killing activity, then the colonies counted from the suspension provide an indirect measurement of the BE of surfaces. Slight variant of this method is also used for quantifying BE. In this variant, bacterial cell count (in CFU) is taken before and after the test.^43,62^ Due to these experimental factors, CFU counting method fails to capture the actual number of dead cells on the substrates. Further, this method requires meticulous control of conditions such as culture medium concentration, and incubation temperature, to eliminate potential errors. However, the colony count method works well with antimicrobial drugs and leaching agents in solutions such as nanoparticles.

The second method is fluorescence staining, as it can visualise both live cells and dead cells under a fluorescence microscope. The viable cells and dead cells are stained by different fluorescence dyes, which get excited by distinct incident light beams.^28,31,53,58,63^ However, this staining will only provide a colour map and not a quantification of exact number of live or dead cells. Cell number quantification is done by interpretation of colour intensity, which creates an ambiguity on the quantification. Sometimes, dual staining of the same cell may lead to erroneous quantification. Disagreement between results obtained using colony counting and fluorescence staining methods has been reported.^87^ Moreover, small field-of-view of the microscopes can lead in to sampling errors depending on the size of the substrate being tested. A large number of images may be required to establish accurate representation of the cell viability on the substrate.

Clearly both CFU counting and fluorescence staining methods does have limitations. Considering the number of factors that can induce uncertainty in bacterial viability results, fluorescence staining method can yield more accurate reading. Main challenge for fluorescence staining method is ensuring the images are representative of the entire substrate. However, this can be mitigated by using proper statistical methods for sample size selection. Another challenge for this method is to post process the images. The colour intensity thresholds must be set for the images, which greatly influence the accuracy of the result. This can be mitigated by using proper control samples to establish threshold settings for live and dead signals.

### Is enumerating bacterial viability under static conditions appropriate?

**Fig. 3:**
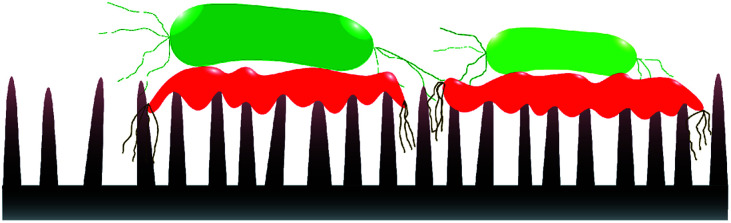
Nano-feature sharpness compromised with lysed cells. Dead cells (symbolised by red colour) are stagnated on nano-features covering the tips. This might prevent subsequent live cells (symbolised by green colour) getting inactivated by the nano features.

Static incubation methods are popular in assessing bactericidal effect of MNSS. Adherence of bacteria onto substrates has been shown to decrease by ∼10 folds in flow conditions compared to static conditions.^88^ Moreover, increasing the fluid shear also reduces bacterial adhesion.^89–92^ Reduction in bacterial adhesion under flow conditions can affect the BE of a substrate, as adhesion of the cell is critical for physical inactivation of cells by micro/nano-scale features. In addition, MNSS have shown higher killing efficiency against motile species of bacteria than against non-motile counterparts.^26,93^ Motility of the bacteria is affected by the external flow. For example, under flow conditions, bacteria uses their extracellular appendages to manure,^42^ or swim upstream.^94^ Further, it has been observed that bacterial growth increases with increasing fluid shear.^95^ Bacterial phenotyping and biofilm density are also known to be influenced by the fluid shear rate.^90^ Behaviour of bacteria in stationary and flowing medium are drastically different. Hence bacteria killing efficiency of MNSS quantified under static conditions is expected to be different under flow conditions as lysed bacteria remain on the surface till the end of the trial.^22,36,38,45,62,63,69,70,74,81,84,96–98^ Nanofeatures may not be able to pierce subsequent bacteria as the tips are covered with remains of previously lysed bacteria. Hence, sharpness, height, and spacing of the micro/nano features are compromised upon dead cell stagnation^99^ as depicted in Fig. 3. Under *in vivo* conditions, dead bacteria adhering onto bioimplants may be ingested by phagocytes^100^ or get advected by the body fluids. Detachment of bacteria from solid substrates under fluid shear signifies that lysed cells can get removed from the substrate under flow conditions.^9,88^ Flowing bacterial suspension through a nanostructured microfluidic channel yielded dead bacteria at the exit, while no bacteria remained on the surface,^101^ indicating that the dead bacteria was flushed away from the nanostructured surface. Hence, BE of MNSS measured under non-flow/static conditions can be underrated compared to that of under actual flow conditions like in urinary catheters, stents, and other industrial devices.

Bactericidal MNSS also have high potential for applications in prosthesis. In order to assess the bacterial viability under actual conditions, such tests should be conducted under representative conditions. Fluid flow in various parts of human body such as cardiovascular system,^102–109^ bladder and urinary system,^110,111^ reproductive system,^112–115^ eyes,^116^ between tissues,^117^*etc.* reported as shown in Table 2. Most of these fluid flows are in laminar flow regime despite the variation in fluid shear. The factor of fluid flow is not captured by currently used static protocols.

**Table 2:** Fluid shear stress inside human body

Location	Wall shear stress (Pa)	Shear rate (s^−1^)	Reference
Urinary tract (ureter and urethra)	0.001–0.5		110
Urethra (during urination)	0.1		111
Bladder	0.1		111
Urinary catheters	0.015–0.03	15.0	149–151
Cardiovascular system	0.01–5.0		102
Eye due to flow of tears	0.005–0.007		116
Heart valves	0.06–37.59		152
Kidney collecting duct cells	0.02–2.0		153
Reproductive system (uterus)	0.1		112
Circulatory system (venous circulation)	0.05–0.6	50.0	103–108
Human umbilical vein	1.0–2.0		113
Peroneal veins	0.009–3.0		114
Placenta	0.05		115
Circulatory system (arterial circulation)	0.4–7.0	650.0	105–109
Large arteries	1.0–4.0		154
Small veins and arterioles	2.0–8.0	2600.0	104 and 108
Capillaries	1.0–2.0		106
Blood vessel walls	0.4–5.0		155
Interstitial fluid (ISF) flow	0.8–3.0		117

Nutrient depletion in static *in vitro* methods may cause some changes to the cell size and envelope.^118^ Supply of nutrients are conditions specific to bioimplants, and these factors may be important in accurately enumerating BE of MNSS generated on a bio-implant, which is better represented in flow conditions.

In other potential applications of MNSS such as in pipelines, marine vessels, ship hulls, and vascular prosthesis does also have a flow of fluids over solid surfaces. Static assays also fails to capture the influence of the overlaying fluid flow on microbial attachment while investigating marine biofouling.^9^ Bacterial growth, movements, and adhesion onto surfaces are affected by presence of fluid flow. Therefore, enumeration of BE should be done under flow conditions that represent the actual conditions.

A common characterisation step after the bacterial attachment protocols is the rinsing of the substrate to remove any bacterial cells that are not attached to the surface, or weakly attached to the surface as discussed previously in this article. The use of fluid flow to remove non-adhered or loosely adhered bacteria from a substrate is disputed^77,119^ as the forces applied may remove the strongly or properly adhered bacteria as well. Hence, substrate rinsing can lead to notable errors in quantifying BE as shear forces exerted on cells under flow can cause the cells to undergo advection. Percentage of bacteria cell detachment from MNSS increases with fluid shear strain.^120^ However, there is no proper definition of what level of strength in adhesion is considered as strong or weak BE quantification experimental design should also test if the fluid flow is equally effective on live and dead bacterial cells on MNSS.

## Bacterial activities under dynamic culture medium

This section reviews bacterial studies performed under non-static test conditions. In such studies, the bacterial adhesion, biofilm formation, and viability have been investigated. However, only a few studies have been reported on bacterial viability on MNSS under flow conditions.

Most of the bacteria are motile, and these motile bacteria uses several mechanisms to obtain motion.^121^ Swarming, swimming, twitching, gliding, and sliding are the types of mechanisms used by motile bacteria. Bacteria having flagella, uses motions of those flagella such as rotation to move in a suspension. Type IV pili are also used by bacteria to move. Induced by external factors, sometimes the bacteria also use passive motion. Interestingly, upstream swimming by *E. coli*,^122–124^ and *P. aeruginosa*^94^ under dynamic conditions is stimulated by various environmental factors such as changes in fluid shear^124^ or surface topography of substrates.^94^ Bacteria are also known to be sensitive to external fluid flow and shear rate.^42^ For example, bacteria swimming near boundaries in external fluid flows have a tendency to swim against the flow and its swimming motion varies with fluid shear rate.^125^

Bacteria live in many different fluidic environments that have different fluid properties such as viscosity. Dependence of bacterial suspension viscosity on bacterial mobility is reported both analytically^126^ and experimentally.^127^ On the other hand, López *et al.* showed that bacterial activities have notable effect on viscosity of the medium containing them.^128^ Moreover, effect of bacterial concentration and bacterial swimming speed on viscosity of the suspension was observed by Sokolov and colleagues.^127^ Interestingly, variation in viscosity with respect to shear rate was reported differentially between motile and non-motile species of bacteria.^129^ Non-Newtonian fluidic behaviour of bacterial suspension has also been observed.^122,129^ They have tested biofilms of *P. aeruginosa*, *Pseudomonas fluorescens*, *K. pneumoniae*, and *Stenotrophomonas maltophilia*, with bacterial species, as both pure and mixed cultures. These studies show that the viscosity of containing medium affect the bacterial motion, as well as the bacterial motion affect the viscosity of the suspension.

Bacterial activities have been studied under various dynamic conditions. Bactericidal effect of titanium nanostructured substrate under mechanical agitations was studied by Diu *et al.*^26^ However, except for mechanical agitation, culture medium was kept static with only initially supplied nutrients. They observed a significant difference in bactericidal effect on *P. aeruginosa* under agitated incubation compared to static incubation, but no such difference was observed with *S. aureus*. Their results indicate that the titanium nanostructured surface was highly effective with motile bacteria but not effective on non-motile bacteria, irrespective of Gram-stain type. Kim *et al.* studied antibacterial properties of nanostructured PMMA surface under constant feeding of nutrients to obtain a biofilm.^130^ The polymeric nanostructured surface induced reduction of bacterial adhesion and viability compared to non-modified surface, but eukaryotic cell growth on the nano-surface was significantly less compared to the control. Thus, it is clear that bacterial viability and biofilm formation are affected by changes in its environment.

### Bacterial activities under flow conditions

**Fig. 4:**
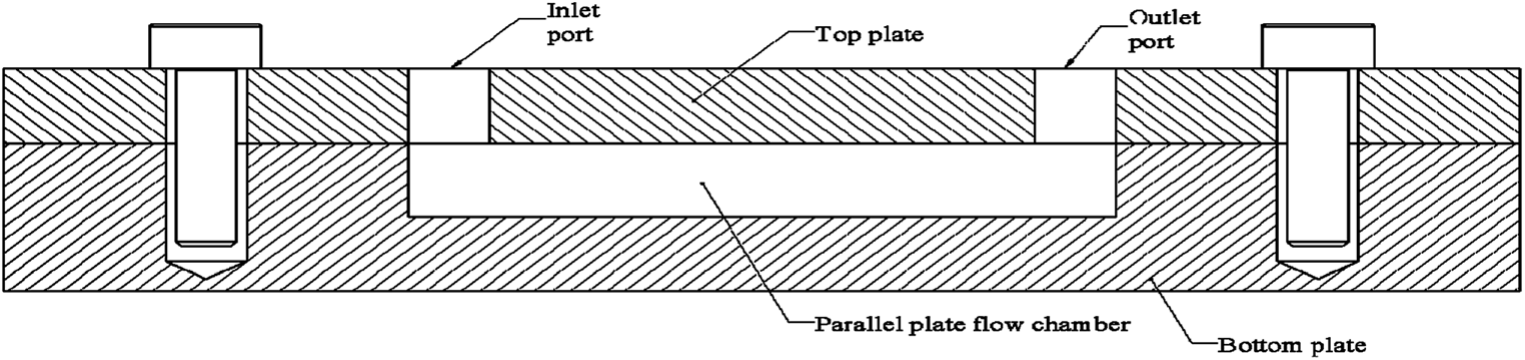
Cross sectional view of PPFC. Fluid enters through the inlet port and flows the chamber situated between two parallel plates. The chamber may be completely on one plate or distributed on the two plates. Surfaces studied for microbial activities are at the chamber on top or bottom, or on both. In most of PPFC designs the plates are made of substrate material used for the study and surface modifications are done to chamber surfaces. In some of the PPFC designs, the subject substrate is integrated into the chamber. Design adaptations were done with transparent plates to enable real-time monitoring of microbial activities inside. On average the chamber is 10 mm wide but has large variations in length. The chamber or the fluid volume typically 0.1–0.5 mm tall.

Microfluidic devices (MFD) have gained popularity in studying cellular activities, as they can efficiently simulate fluid shear stresses with high controllability.^9,131,132^ MFDs has been used to study pathogenic bacteria,^101,133^ marine bacteria,^9^ fungi,^134^ blood cells,^135^ and mammalian cells,^131,136^ under fluid flow conditions. Parallel plate flow cell (PPFC) which is a type of MFD, has been widely used for testing bacterial activities under flow conditions. The PPFC design has two parallel plates with a gap between them for the fluid to flow as depicted in Fig. 4. PPFC principal is used for developing devices for microbial studies in a wide range of scales, as well as it can be scaled into macroscale devices too. Next section presents a summary of MFDs used for bacterial adhesion, biofilm formation, and viability studies on solid surfaces.

### MFDs used for bacterial studies

Myriad studies are performed on bacteria using MFDs of various sizes and configurations. These MFDs are mainly used to induce shear stress on the adhered bacterial cells. Table 3 presents a summary of MFDs used for bacterial studies in recent literature. Values of shear stress and shear rate were calculated from the available data, where wall shear stress or shear rate was not presented in the respective literature. Eqn (1) was used to calculate the wall shear in devices with rectangular cross section. Eqn (2) was used to calculate either wall shear stress or shear rate when the other is reported in the literature or calculated from eqn (1). In cases where fluid shear is calculated, it was assumed the dynamic viscosity of the fluid flowing in the device is equal to that of water at 25 °C.1
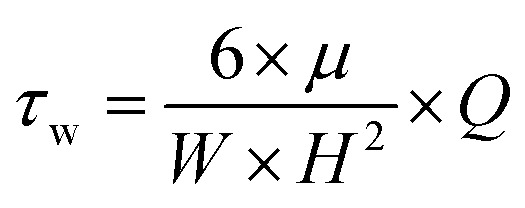

2 *τ*_w_ = *μ* × *γ*

Here, *τ*_w_ is the wall shear stress in Pa, *μ* is the dynamic viscosity of the fluid in Pa s, *W* is the width of the fluid channel in m, *H* is the height of the fluid channel in m, *Q* is fluid flowrate in m^3^ s^−1^, and *γ* is the shear rate in s^−1^.

**Table 3:** Wall shear stress levels and shear rates used in microfluidic devices for microbial studies under flow^a^

X sec.	Flowrate (ml min^−1^)	Wall shear stress (Pa)	Shear rate (s^−1^)	Substrate material	Microbe/s	Ref.
Rt	0.5	0.03–4.3	3981.5^b^	PMMA, glass, PDMS	*Cobetia marina*	9
Rt	1	0.007^b^	6.0	Ti	*S. aureus*	61
Rt	0.01–0.1	0.17–1.68	1527.3^b^	PDMS	*E. coli*	77
Rt	0.97	0.138^b^	15	Coated glass	*E. coli*, *S. epidermidis*	83
Rt	200	0.184^b^	37.0	Aluminium	*S. aureus*, *E. coli*	88
Rt		0.006^b^–0.24^b^	50–2000	PMMA	*S. epidermidis*	89
Rt		0.001–0.2	224.7^b^	Glass	*P. aeruginosa*, *E. coli*, *S. epidermidis*, *P. putida*	91
Rt	60–600	0.005–0.07	82^b^	PDMS/glass	*E. coli*	92
Rt	0.115	1	1250^b^	PMMA	*P. aeruginosa*	94
Rt	0.006	0.237^b^	266.7^b^	Silicon	*P. aeruginosa*	101
Rt	1–10	0.007^b^–0.07^b^	6.0–60.0	Silicon	*S. epidermidis*, *S. aureus*	120
Rt	60–480	0.005–0.056	65.7^b^	Polymers	*E. coli*	133
Rt	0.01	0.001^b^	1.4^b^	PDMS, glass, Ti	*E. coli*	142
Rt	0.00067–0.0651	0.01–1	1195.6^b^	PEO, PLLA, PA, PDMS, PS	*E. coli*	137
SQ		0.00568–0.01135	12.8^b^	Glass	*Acinetobacter* sp.	138
Rt		0.002–700	787 000^b^	PDMS	*Cobetia marina*	139
Rt	16 000–83 000	1.6–24.8	1666.8^b^	Coated substrates	Marine biofilm	140
Rt	0.0004–0.0005	0.0068–0.0852	95.7^b^	PDMS	*P. aeruginosa*	141
Rt	0.3–5	0.0003^b^–0.004^b^	5^b^	Ti, SS	*Lactobacillus delbrueckii*	146
Rt	5	0.007^b^	7.8^b^	Ti, SS	*E. coli*	147
Rt	0.021–1.2	0.002–0.079	2.3–116.1	Ti	*S. sanguis*	148
Rt	0.0035–0.17	0.043^b^–2.101^b^	2361.1^b^	PDMS	*P. aeruginosa*	156
Rt	0.5–10.0	0.002^b^–0.044^b^	49.6^b^	PDMS		157
Rt	0.0001–0.08	0.01–10	50–10 000	PDMS	*P. aeruginosa*	158
Rt	100	1.971^b^	2.0–40.0	PDMS	*E. coli*	159
Rt	1.5	0.014^b^	10	PEO-coated glass	*S. epidermidis*, *S. aureus*, *S. salivarius*, *E. coli*, *P. aeruginosa*	160
Rt	2.0	0.007^b^	7.4^b^	Glass	*P. aeruginosa*	161
SC	0.42	0.417	468.5^b^	PMMA	*E. coli*	162
SC	0.41667	0.6	674.2^b^	Perspex	*E. coli*	163
Rt	60–480	0.005^b^–0.042^b^	7.0–80.0	Peptide coated glass	*E. coli*	164
Rt	0.055	0.0001^b^	0.1^b^	PMMA	*P. aeruginosa*	165
Rt	25	0.163^b^	175.0	Glass	*S. mutans*, *S. sanguis*, *S. mitis*, *S. salivarius*	166
Rt	30–100	7.42^b^–24.72^b^	28 000^b^	PMMA	*S. epidermidis*	167

a Rt – rectangular; SQ – square; SC – semi-circular.

b Calculated from available data.

Most of these MFDs were fabricated from polymeric materials. In these devices, the substrate to be tested of different material types are integrated into the device. MFDs with rectangular cross section were popular due to its ability to provide uniform wall shear distribution as well as the ability of incorporating a transparent inspection window to the device. Large variation of wall shear stress or shear rate has been used in microfluidic bacterial studies. Most of the studies were aimed at studying bacterial activities under conditions related to biomedical applications, while some were focused on studying the same under industrial conditions, such as bacterial adhesion on ship hulls. Though, the aim of study was to emulate fluid flow related to biomedical applications, many of these studies have deviated from fluid shear stress ranges appropriate for the intended scenario. This disparity is evident from Tables 2 and 3. There was no MFD found in literature that can be used to hold a solid substrate inside with repeatability or reusability. The existing designs requires either micro/nanostructure fabricated on the device surface, preferably made using a polymer such as PDMS, or the surface-modified substrate encapsulated in the device made with a polymer. Mostly, micro/nanostructures on metallic substrates are tested due to the high potential of applications. The existing MFD designs creates challenges for testing surface-modified metallic substrates. Therefore, designing a suitable MFD to exert predetermined level of fluid shear on a surface-modified solid substrate is useful for antibacterial surface development.

### Bacterial activities on non-surface modified substrates

In this section, studies reported on bacterial activities under flow conditions on untreated solid substrates are presented. Adhesion of bacteria onto solid substrates under flow conditions of bacterial suspension has been studied. According to Duddridge *et al.*, adhesion of *P. fluorescens* on stainless steel substrate has been reduced with the increase of fluid shear stress level.^50^ Further, they reported that adhesion was highly dependent on the time of exposure as well as initial bacterial cell concentration. Wang and team studied the adhesion of *S. epidermidis*, *P. aeruginosa*, and *E. coli* species on glass and octadecyl trichlorosilane (OTS) modified glass substrates with varying fluid shear, and observed a similar trend of decreased adhesion with increasing shear stresses.^91^ Reduction in *E. coli* adhesion on glass or PDMS substrate with increasing fluid shear stress is also observed by Moreira and the co-workers.^92^ Wang *et al.* observed a trend in bacteria to adhere as single cells, or form smaller clusters, when the fluid velocity increased.^91^ Effect of hydrophobicity on adhesion under flow condition was emphasised.^92^ Katsikogianni *et al.* studied adhesion of bacteria on to hydrolysed glass substrates with varying flowrates and observed adhesion of bacteria on the hydrophobic substrates was significantly reduced with increasing shear rate.^89^ Wang *et al.* have observed 900% increase in *P. aeruginosa* and 100% increase in *P. putida* attachment on hydrophobic substrate than hydrophilic counterpart under flow conditions.^91^ However, *E. coli* attachment was 30% less in hydrophobic surface. Ponmozhi and team studied adhesion of *E. coli* on several polymeric substrates.^137^ Glass tubular and rectangular cross sectioned flow has been used to study the adhesion of *Acinetobacter* spp. on PMMA surfaces.^138^ In this study, no significant effect of shear stress level on bacterial adhesion was observed. However, a temporal study of bacterial adhesion has shown an increase in number of bacterial cells on the surface by means of new adhesions to the surface and stacking of bacteria on existing clumps. Notably, this study had used fluid shear of 0.00568 to 0.01135 Pa, which is much lower compared to the mean fluid shear range (0.39–21.02 Pa) used in other studies. These lower levels of fluid shear as well as narrow range for the shear, may have caused only subtle variations. Fluid shear, length of exposure, bacterial concentration affects the bacterial adhesion onto solid substrates of different forms, as well as the substrate surface conditions.

Effect of fluid flow on detaching the adhered bacteria has been studied. Arpa-Sancet and colleagues reported that detachment of *Cobetia marina* bacteria adhered onto a polymer coated glass substrate has occurred only after reaching a threshold shear stress (∼5 Pa).^139^ However, another research showed that even though the rate of bacterial transport to the surface increased due to increased flowrate, it has not increased at-par with the rate of removal of bacteria from the surface.^50^ A similar claim has been presented by Wang *et al.* as well.^91^ In (ref. 50 and 91) the bacterial suspension flow was continuous, where in ref. 139 the bacteria were preincubated on the substrate and subjected to flow afterwards. Therefore, new adhesions were not possible in the latter study, which explains the differences in observations. Increasing the fluid shear stress has caused an increase in shear-induced removal of biofilm from solid substrate coated with anti-biofouling polymer layer.^140^ Similarly, increasing the fluid shear hindered *P. aeruginosa* biofilm formation on PDMS polymer substrates.^141^ It is apparent that bacteria get removed from the attached substrates under certain levels of fluid shear, however the values are not clear for each species.

Many researchers considered gravity as an influential factor on bacterial adhesion onto surfaces. Bacterial adhesion tests performed with substrates held upright and upside-down under flow conditions had comparatively lower adhesion on upside-down surfaces.^138,142–144^ Similarly, under non-flowing condition, bacterial adhesion on upside-down substrate has been lower than that of upright substrate.^26^ Li *et al.* studied the adhesion of *S. aureus* on glass slides held at top and bottom in a PPFC under laminar flow and observed adhesion on bottom surface increased over the time, while that on top surface reached a maximum soon after start of the flow.^144^ Moreover, they observed reduction in initial bacterial deposition on the bottom surface with increasing flowrate, while bacterial deposition on top surface was slightly increased with increasing flowrate. These observations confirmed the gravitational effect on bacterial adhesion. However, higher adhesion on top surface is possible under specific conditions as adhesion depends on other factors such as flow velocity, cell diffusion rate, bacterial motility, and suspension medium. For example, higher flow velocities can cause higher advection of bacterial cells than diffusion, hence a sedimentation effect could not be observable.

Some studies were conducted to examine bacterial adhesion and biofilm formation under industrial conditions. Salta *et al.* used the principle of PPFC to develop a flow chamber to study bacterial attachment and biofilm formation on glass and polymer surfaces under service conditions of a ship hull.^9^ This PPFC had four chambers in tandem with reduced chamber height in the direction of the flow to obtain different shear stress level in same bacterial suspension flow representing four different ship velocities. In agreement with other studies they also observed that the bacterial adhesion was significantly reduced with increasing shear stress levels. However, the flow chambers in tandem raises the concern of reducing the bacterial concentration in the fluid, as the bacterial adheres to the initial chambers. Mulansky *et al.*^145^ studied the *E. coli* biofilm formation under laminar flow and the effect of substrate surface roughness on biofilm formation. Their results show an increase in surface roughness from 9.6 to 43 nm which caused an increase in biofilm coverage on the surface from 18% to 95%. *P. fluorescens* biofilm formation on stainless steel substrate under flow conditions was reduced significantly with increasing fluid shear.^50^ These studied indicate that biofilm formation is clearly affected by fluid flow. Furthermore, these studies had used various devices that were developed based on the PPFC principle. This shows the suitability and adoptability of PPFC for microbial studies from micro to macro scale.

### Bacterial activity on MNSS under flow conditions

This section is focused on studies conducted for testing bacterial activities on micro or nanostructured surfaces under flowing media.

Adhesion of *E. coli* on polymer MNSS was affected by shear stress level, with a 60% reduction in adhesion at 0.05 Pa shear rate and a maximum reduction of 90% at 0.025 Pa.^133^ They further observed that the adhesion of bacteria onto nanostructured surface was reduced under shear stress compared to a smooth surface, and the rate of reduction was positively affected by the exerted level of shear. Adhesion of *E. coli* and *S. epidermidis* on a silicon coated substrate under static and dynamic conditions using a PPFC has been studied by Meier and team with flow parameters set to emulate shear rate inside a urinary catheter.^83^ Their results suggest that adhesion of bacteria is dependent on the types of fluid and the substrate. Detachment of *S. aureus* and *S. epidermidis* bacteria from nanostructured surface has been increased by 10 folds when the shear rate was increased by 10 folds.^120^ In the same study, the nano-topography of the substrate has significantly reduced the adhesion forces between bacterial cell and the surface. Hydrophobic nano-topographic aluminium surfaces were seen with significantly less adhesion of *S. aureus* and *E. coli* compared to the hydrophilic surface with same nano-topography under both static and flow conditions.^88^ The adhesion forces between bacterial cells and silicon substrate varied with nanotopography^120^ but the adhesion forces between bacterial cells and aluminium substrates^88^ remained same with varying nanotopography. Graham *et al.* studied adhesion of *E. coli* on microtopographic PDMS, glass and titanium substrates, and observed increased adhesion under flow compared to the static condition.^142^ The stiffness of the surface also affects the retention of *E. coli* bacteria on a PDMS surface.^77^ Higher retention of bacteria under varying fluid shear was seen with less rigid substrate despite similar adhesion of ∼90% and ∼95% under 0.84 Pa and 1.68 Pa shear stress levels. However, they observed that there is no significant difference in adhesion between soft and rigid substrates at low shear stress level of 0.17 Pa. It can be concluded that flow reduces bacterial attachment and increases detachment from MNSS. The substrates' surface characteristics such as surface hydrophobicity, stiffness, material type and micro/nano-topography does have a prominent influence on attachment, and detachment of bacterial cells on MNSS under flow conditions.

Biofilm formation on MNSS under flow is another aspect that has been extensively studied. A flow-cell based on the principle of PPFC was developed by Schlegel and team to study *Lactobacillus delbrueckii lactis* bacteria adhesion and biofilm formation on microstructured stainless steel and titanium substrata.^146^ There was no significant difference in biofilm formation between non-surface-modified titanium and non-surface modified stainless-steel substrata. Moreover, there was no significant difference in biofilm production on the surface-modified titanium and surface-modified stainless-steel substrata. However, in this experiment, the flow used was of an intermittent type with 20% of flow-time where the medium was stationary for 80% of the time. Therefore, the effect of flow could be undermined by the static phases of the flow cycle. Biofilm formation of *E. coli* strain on surface-modified stainless steel and titanium substrata under flow conditions has also been studied by Kleine *et al.* using a custom designed flow-cell following PPFC principle.^147^ Notably, continuous monitoring of the biofilm was done using confocal laser scanning microscope (CLSM). The difference in biofilm coverage between the samples of stainless steel and titanium with the same topography was not significant. However, the titanium substrates with different surface-topographies showed significantly different biofilm formation. This study also employed a non-continuous, 20% flow cycle. Several types of nanostructured surfaces under various flow conditions were tested for bacterial adhesion by Bierbaum *et al.*^148^ All four types of nanostructured substrates showed decreased bacterial biofilm coverage on the surface under flow compared to the static medium by ∼50–2800 folds. By increasing the fluid velocity, three of the substrates showed reduction in biofilm coverage on surface. While on the rougher surface there was an increase in biofilm coverage, indicating that in addition to the flow velocity, surface topography also affects the biofilm formation. In general, MNSS caused a reduction in biofilm formation, subjected to the effect of surface-topography and flow parameters.

Only few studies were found on bacterial viability on MNSS under flow conditions. Li *et al.* fabricated micropillars on silicon substrates using reactive ion etching followed by metal-assisted chemical etching, they created nanoscale lateral spikes on those micro-scale pillars, and had tested the viability of *E. coli* under flow using a PPFC based MFD.^99^ Markedly, the fabricated micropillars are patterned, and the sharp spikes are parallel to the flow direction, where other studies have used the micro/nanoscale features perpendicular to the flow direction. There was a significant reduction in number of viable bacteria (CFU) in downstream with a killing efficiency of 80%. The authors hypothesised that an increase in the flow velocity could increase the killing efficiency of substrate due to the impact force on bacterial cells during collision with micropillars. In another study, a microfluidic channel containing a nanostructured black silicon surface was used to test bactericidal effect under flow.^101^ They claimed a BE closed to 99% on *P. aeruginosa* after 5 cycles. Notably this work has used an intermittent flow with only 7% flow time. The substrate was hanged upside down inside the microfluidic chamber. Moreover, fluid flow velocity of 0.00033 m s^−1^ is very low, and it is possible that diffusive transport is dominant than advective transport of bacteria in the flow. Therefore, a higher number of bacteria settling to the bottom of the flow-cell can be expected. Several studies have shown the effect of gravity that relates to lower adhesion of bacteria on the top surface of a flow-cell than on the bottom surface as discussed previously.^138,142^*S. aureus* tested on titanium substrate with nano-pillars and nano-pores under flow with shear rate of 6 s^−1^ (≈0.006 Pa) resulted in low BE of approximately 15% and 23% respectively.^61^ Noticeably, the nano-pillars were very thin with 10 nm diameter, 2 μm height and sparsely distributed with 2 μm spacing. Under static conditions, the BE of MNSS depends on bacterial species, micro/nanotopography of the surface, and arguably the substrate surface hydrophobicity. These factors may or may not affect the BE under flow conditions and the effects might not be the same. For example, adhesion of bacteria onto MNSS under flow conditions is different from MNSS under static conditions. Influence of fluid flow on BE of MNSS has not been sufficiently explored, and there remain many research questions unanswered.

It has been established though that bacterial adhesion onto MNSS is dependent on fluid shear and fluid flowrate. Moreover, as discussed earlier, attachment of cells onto substrate is required for cell inactivation by micro/nano-scale features. Under static conditions, parameters such as bacterial concentration affects the number of bacterial cells attaching to a solid surface. Effects of these on bactericidal action of MNSS under flow conditions has not been studied.

**Fig. 5:**
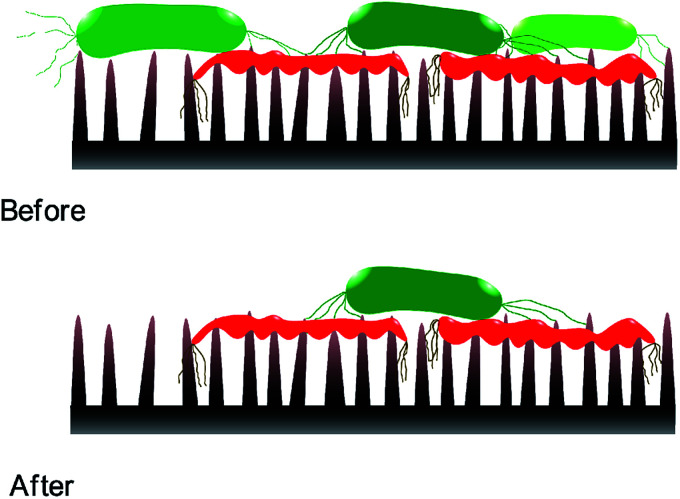
Rinsing the MNSS substrate before bacterial viability quantification is a common step in currently used protocols. Dead bacterial cells (symbolized by red colour) are pierced by nanoscale features on the surface while live cells are attached to the surface by physiochemical interactions. A sufficient fluid shear can overcome the adhesion strength of live cells (symbolized by green colour) which thus causes the cells to detach from the surface. Therefore, it is highly likely to have more live cells get removed than dead cells from the surface, which may result in an erroneous quantification of the bactericidal efficacy of the surface.

In addition, it has been shown that fluid shear can cause detachment of adhered bacteria from a solid substrate. Bacterial cells have also been pierced by the nanoscale features on MNSS. Given these facts, it can be hypothesised that adhesion strength of live and dead bacteria is different, and therefore when subjected to a flow such as rinsing of substrate, the live cells may get advected more than the dead cells as depicted in Fig. 5.

## Conclusion

MNSS with different topographies have been investigated against bacterial cells. However, only certain nanoscale topographies such as nanopillars, nanowires, nanospikes, nanocrystals have shown a bactericidal effect whereas other have contributed towards a lower bactericidal or antibiofouling effect.

Though, traditionally believed that MNSS are less effective in killing Gram-positive species of bacteria, there is sufficient literature against that. More tests are needed to know that the bactericidal effect of MNSS is based on the Gram-stain type of bacteria. Influence of substrate surface hydrophobicity on BE of MNSS cannot be generalised too. However, it can be concluded that effect of hydrophobicity on BE of MNSS varies with the bacterial species tested on the substrate.

Accurate quantification of BE of a MNSS requires the number of both live cells and dead cells on the substrate. In view of this, the fluorescence staining method can provide more accurate quantification results than standard plate colony counting method.

Potential applications of MNSS in medical sector as well as in industrial sector does have some sort of overlaying fluid flow. However, the current method adopted to quantify the bacteria killing efficiency of solid surfaces are done under static conditions. Nevertheless, adhesion of bacteria onto surface, and mobility of bacteria are different under flow conditions than static conditions along with some other aspects such as bacteria phenotyping. Further, bacterial detachment from surfaces is increased under flow conditions. On the other hand, stagnation of dead cells on MNSS occurs under static conditions, which can result in false killing efficiency enumeration of MNSS. Therefore, the absence of flow in the current static *in vitro* bacterial viability quantification on MNSS assays differs from the conditions of many of the potential applications. Fluid flow is application dependent and bactericidal effect should be tested under conditions appropriate for the application. Bacterial attachment is reduced and detachment of adhered bacteria from MNSS increased under flow conditions. Adherence of bacteria to the substrate is essential for lysing bacteria by the nano-scale features on the surface. Reduction of adhesion can be beneficial in reducing biofilm formation, but it can adversely affect the bactericidal property as contact to surface is required for lysing bacteria. The effect of reduced bacterial adhesion under flow conditions and the further effect on bacteria lysing capability of MNSS has not been reported. Given all these, it can be hypothesised that the bactericidal effect of MNSS is reduced under flow conditions.

Only few studies on BE of MNSS under flow conditions has been reported. This provides lot of research opportunities, as there are many factors contributing for the BE of a surface under flow conditions. Hizal *et al.*^88^ stated that the antibacterial effect of MNSS with culture medium under flow has not been previously studied, and no further studies have been conducted on this topic since then. Gravity is influential on bacterial adhesion under low flow conditions. The influence of gravity and sedimentation on bacterial adhesion and lysing with MNSS has also not been systematically explored. Motility of bacteria is also found to be influential on bactericidal effect of MNSS, but it is also not yet explained how bacterial motility can affect bactericidal effect of substrate under flow.

## Conflicts of interest

There are no conflicts to declare.

## Supplementary Material
